# Predictive Factors, Treatment, and Outcomes of Coagulase-Negative Staphylococcal Peritonitis in Malaysian Peritoneal Dialysis Patients: A Single-Center Study

**DOI:** 10.1155/2022/8985178

**Published:** 2022-04-11

**Authors:** Siew Yan Lau, Boon Cheak Bee, Hin-Seng Wong, Marhanis Salihah Omar, Norazlah Bahari

**Affiliations:** ^1^Department of Pharmacy, Selayang Hospital, Selayang, Malaysia; ^2^Department of Nephrology, Selayang Hospital, Selayang, Malaysia; ^3^Clinical Research Center, Selayang Hospital, Selayang, Malaysia; ^4^Faculty of Pharmacy, The National University of Malaysia, Bandar Baru Bangi, Kuala Lumpur, Malaysia; ^5^Department of Pathology, Selayang Hospital, Selayang, Malaysia

## Abstract

**Aims:**

Coagulase-negative *Staphylococci* (CoNS) are frequently isolated in peritoneal dialysis (PD)-related peritonitis with a high rate of relapse and repeat peritonitis after initial response to antimicrobials. The optimal treatment regimen for CoNS peritonitis remains debatable. Hence, this study aimed to describe the clinical and microbiologic characteristics of CoNS peritonitis in a PD center and determine predictive factors influencing the outcomes.

**Methods:**

All cases of CoNS peritonitis in Selayang Hospital between 2011 and 2019 were reviewed retrospectively.

**Results:**

A total of 906 episodes of peritonitis were recorded; 140 episodes (15%) in 98 patients were caused by CoNS. The oxacillin and gentamicin resistance rates were 47% and 46%, respectively. The overall primary response rate was 90%, and the complete cure rate was 79%. Patients with concomitant exit-site infection (odds ratio (OR) 0.06, 95% confidence interval (CI) 0.01 to 0.40, *P* < 0.01) and history of recent systemic antibiotic use (OR 0.04, 95% CI 0.01 to 0.82, *P*=0.04) were less likely to achieve primary response. CoNS episodes that were treated with beta-lactam-based or vancomycin-based therapy had a similar primary response rate and complete cure rate. The rates of relapse and repeat were 12% and 16%, respectively. Relapsed episodes (OR 0.35, 95% CI 0.13 to 0.97, *P*=0.04) had a significantly lower complete cure rate than the first episodes.

**Conclusion:**

Relapsed CoNS peritonitis was common and was associated with worse outcomes than the first episode of CoNS peritonitis. Oxacillin resistance was common, but the treatment outcome remained favourable when a beta-lactam-based regimen was used as empirical therapy.

## 1. Introduction

Peritonitis remains a major complication in peritoneal dialysis (PD) patients. Severe and prolonged peritonitis may result in structural and functional alterations of the peritoneal membrane and eventually peritoneal membrane failure [1]. It is one of the many causes of technique failure among PD patients [2]. Peritonitis does not only increase the risk of infection-related mortality but has also been associated with an increased risk of all-cause mortality and cardiovascular mortality [3]. In many PD centers, Gram-positive bacteria are the most common organisms causing PD-related peritonitis with coagulase-negative *Staphylococci* (CoNS) contributing to nearly half of the cases [4, 5].

Most of the patients with CoNS peritonitis have mild clinical symptoms and respond rapidly to antibiotic therapy [6, 7]. Two weeks of an effective antibiotic are recommended to achieve a complete cure [1]. However, CoNS peritonitis has the highest relapsed and repeat rates compared to peritonitis caused by other organisms [8]. Furthermore, oxacillin resistance among CoNS isolates is common [6, 9] and has been associated with the nonresolution of CoNS peritonitis [7]. Hence, the optimal treatment regimen for CoNS peritonitis is debatable and the use of vancomycin as the initial treatment of CoNS peritonitis remains controversial [6, 7].

CoNS peritonitis, especially those caused by *Staphylococcus epidermidis*, often results in biofilm formation in the PD catheter [7], which has been associated with relapsed and repeat CoNS peritonitis [8]. It has been demonstrated that combination antibiotic therapy reduced the formation of biofilm by CoNS isolates [10] and a 3-week course of single antibiotic has been reported to be associated with a higher resolution rate compared to the conventional 2-week course of antibiotic for the treatment of relapsed and repeat CoNS peritonitis [9]. However, there are no published studies on the effectiveness of a 3-week dual antibiotic therapy in treating CoNS peritonitis. Hence, the current study aimed to describe the clinical and microbiologic characteristics, treatment, and factors influencing the outcomes of CoNS peritonitis among Malaysian PD patients.

## 2. Methods

This was a retrospective study conducted in a PD center at a tertiary hospital in Malaysia. All CoNS peritonitis episodes that occurred between 1 January, 2011 and 31 December, 2019 in adult PD patients (≥18 years old) in our center were identified from our center's PD peritonitis registry. Patients who met any of the following criteria were excluded from the study: (i) PD effluent yielded CoNS but did not fulfill the criteria of PD peritonitis; (ii) PD effluent yielded more than 1 microorganism; (iii) patients with incomplete clinical data. The following information was retrieved from the hospital electronic medical record: (i) demographic data; (ii) dialysis vintage and modalities; (iii) clinical characteristics at presentation; (iv) history of peritonitis-related infection, recent hospitalization, and antibiotics use (within 3 months before the onset of peritonitis symptoms); (v) peritonitis-episode date, antibiotic susceptibility, antibiotic regimen (empirical and definitive), and clinical outcomes. All patients were followed up for at least 12 months from their last episode of CoNS peritonitis. The overall rate of peritonitis was calculated for this 9-year period and expressed as the number of episodes per patient-year of treatment [1]. Relapsed and repeat peritonitis was recorded as a separate peritonitis episode.

Gram stain, microscopy for cell count, and culture on blood and MacConkey agar at 37°C for 48 hours were performed on the dialysate samples. Samples were inoculated into aerobic and anaerobic blood culture broth. Subsequently, samples were monitored continuously using the automated BACTECTM 9240 Instrumented Blood Culture System, 5 days for bacterial growth and 14 days for fungal growth. Samples were also sent in a sterile screw-capped container and directly inoculated on a culture agar plate. If Gram-positive cocci were isolated, a coagulase test would be performed. Antibiotic sensitivities were determined by testing the cultures via the disc-diffusion method. Strains presenting intermediate values were considered resistant.

Peritonitis was diagnosed when at least two of the following criteria were met: (i) clinical features suggesting peritonitis, that is, abdominal pain and/or cloudy peritoneal dialysate; (ii) peritoneal dialysate white cell count >100/*μ*L (after a dwell time of at least 2 hours), with more than 50% polymorphonuclear neutrophils; (iii) at least 1 positive peritoneal dialysate culture. The patient was diagnosed with CoNS peritonitis when CoNS were isolated from the peritoneal dialysate samples [1]. Exit-site infection was diagnosed when there was purulent discharge, with or without erythema of the skin around the catheter-epidermal interface [11]. Initial antibiotics for peritonitis were generally intraperitoneal administration of vancomycin or cefazolin plus an aminoglycoside and ceftazidime. The antibiotic regimen was changed to vancomycin plus gentamicin when the diagnosis of oxacillin-resistant strain was ascertained. The dosage of antibiotics is as follows:

- *Cefazolin and Ceftazidime*. A loading dose of 500 mg/L and a maintenance dose of 125 mg/L were administered in all PD exchanges
- *Gentamicin*. A loading dose of 8 mg/L and a maintenance dose of 4 mg/L were administered in all PD exchanges
- *Vancomycin*. A loading dose of 1000 mg/L and a maintenance dose of 25 mg/L were administered in all PD exchanges [12, 13]

Serum gentamicin and vancomycin levels were taken on day 4 and repeated if necessary. The dosage of gentamicin and vancomycin was reduced by 25% if the measured serum levels exceeded 2 mg/L and 20 mg/L for gentamicin and vancomycin, respectively. If patients complain of impaired hearing, tinnitus, or other symptoms of ototoxicity, they would be referred for a further hearing assessment.

In this study, the primary response referred to the resolution of abdominal pain, clearing of the peritoneal dialysate, and peritoneal effluent neutrophil count <100/*μ*L on day 10 with antibiotics alone. Complete cure was defined as complete resolution of peritonitis by antibiotics alone without relapse or recurrence within 4 weeks of completion of therapy. The patient was diagnosed with relapsed peritonitis when an episode occurred within 4 weeks after completion of therapy of a prior episode with the same CoNS species or 1 sterile episode. Repeat peritonitis referred to an episode that occurs more than 4 weeks after completion of therapy of a prior episode with the same CoNS species. Death was defined as the death of a patient with active peritonitis or of a patient who had an episode within the previous 2 weeks [7, 13].

Results were expressed as frequencies and percentages for categorical variables, mean ± standard deviation for continuous normally distributed variables, and median (interquartile range) for continuous non-normally distributed variables. Dichotomous and categorical data were compared using chi-square or Fisher's exact tests. Backward likelihood ratio multiple logistic regression analysis was used to assess the independent predictive factors for the outcomes of CoNS peritonitis. Variables with *P* < 0.25 in univariate analyses were included in the multivariate analysis. The Hosmer–Lemeshow test was used to assess the fit of logistic regression models. Data were analyzed using the software package PASW Statistics for Windows (Version 24.0: IBM Corp, Armonk, NY, USA). Odds ratios (ORs) with 95% confidence intervals (CIs) were calculated. *P* values less than 0.05 were considered statistically significant.

Ethics approval was obtained from the National University of Malaysia (UKM) Human Research Ethics Committee (UKM PPI/111/8/JEP-2018-112) and the Ministry of Health Medical Research Ethics Committee, Malaysia (NMRR-17-2696-38761) prior to the commencement of the study.

## 3. Results

Between 2011 and 2019, 906 episodes of peritonitis were diagnosed, with an overall peritonitis rate of 0.23 episodes per patient-year of treatment. During the study period, 140 episodes of CoNS peritonitis occurred in 98 PD patients. The overall CoNS peritonitis rate over the 9-year period was 0.04 episodes per patient-year of treatment. Ninety-five patients presented with the first episode of CoNS peritonitis and 3 patients with the repeat CoNS peritonitis when they were first recruited during the study period. Seventy-four patients had 1 episode, 13 patients had 2 episodes, 8 patients had 3 episodes, and 3 patients had more than 3 episodes of CoNS peritonitis during the study period. Of 140 CoNS peritonitis episodes, 95 (68%) episodes were new episodes, 15 (11%) were relapses, and 30 (21%) were repeat. Two patients with exit-site infection had CoNS isolated in peritoneal dialysate samples but did not fulfill the criteria of PD peritonitis (dialysate cell count was zero and did not have symptoms of peritonitis).

Demographic and clinical data from the first episode of CoNS peritonitis are summarized in Table 1. Forty-four (46%) patients had a history of hospital admission and 27 (28%) patients had a history of systemic antibiotic therapy within 3 months of the onset of CoNS peritonitis. Among these 27 patients, 14 (52%) patients received antibiotics for recent peritonitis episodes. The median time between symptoms onset and presentation to the hospital was 2 days. In 9 (10%) patients, there was a concomitant exit-site infection, whereas 35 (37%) patients had a history of PD-related peritonitis.

Antimicrobial susceptibility data were available for 127 out of 140 episodes. The *in vitro* antibiotic susceptibility of CoNS isolates was as follows: (i) 67 (53%) CoNS were oxacillin-susceptible strains; (ii) 69 (54%) CoNS were susceptible to gentamicin; (iii) for vancomycin, E-test was performed only in the year 2011 and 2012, and all isolates (*N* = 24) were susceptible to vancomycin. When subanalysis was performed to compare antibiotic resistance between oxacillin-resistant and oxacillin-susceptible strains, resistance to other antibiotics was found to be higher among oxacillin-resistant strains (Table 2). Patients with the history of recent hospitalization had a significantly higher risk of being infected with oxacillin-resistant strains (56% *vs* 38%, *P*=0.04), whereas gentamicin-resistant strains were more commonly found in patients with the history of recent hospitalization (55% *vs* 36%, *P*=0.04) and recent systemic antibiotic use (57% *vs* 39%, *P*=0.04). Other patient characteristics were not associated with the risk of oxacillin and gentamicin resistance (Table 3).

Cefazolin was the most common empirical antibiotic given for Gram-positive coverage in both first (68%) and repeat (69%) CoNS peritonitis, whereas vancomycin was the most common empirical antibiotic given for Gram-positive coverage in relapsed cases (53%) (Table 4). Vancomycin was also the most common antibiotic (59%) prescribed as definitive therapy for all types of CoNS peritonitis while gentamicin was the most common aminoglycoside used in treating peritonitis, in both empirical (95%) and definitive (86%) therapy regimens. Other less commonly used antibiotics in CoNS peritonitis include piperacillin/tazobactam, cefepime, meropenem, trimethoprim/sulfamethoxazole, rifampicin, and linezolid.

Of a total of 140 episodes, 81 episodes (58%) involved modification of the antibiotic regimen after a median period of 5 days, where 69 cases (85%) were converted from beta-lactam/aminoglycoside to vancomycin/aminoglycoside combination therapy due to oxacillin-resistance (38 cases (47%)), poor clinical response (9 cases (11%)), and the use of vancomycin as definitive therapy for oxacillin-susceptible CoNS (22 cases (27%)). Overall, the total duration of antibiotics was 21 days for beta-lactams and vancomycin and 14 days for aminoglycosides.

The overall primary response rate was 90%, whereas the overall complete cure rate was 79%. The rate of primary response was highest among those who had the first episode of CoNS peritonitis, followed by repeat and relapsed peritonitis (94%, 87%, and 73%, respectively, overall chi-square test, *P*=0.04) (Table 5). Similarly, the complete cure rate was higher in those who had first CoNS peritonitis compared to those with repeat or relapsed CoNS peritonitis (87%, 67%, and 47%, respectively, overall chi-square test, *P*=0.04). The rates of relapse and repeat were 12% (17 episodes) and 16% (23 episodes), respectively. The rate of relapsed peritonitis was lowest in patients who had the first episode of CoNS peritonitis (7%) compared to those who had relapsed (33%) or repeat peritonitis (25%, overall chi-square test, *P*=0.01). The average duration for relapsed peritonitis to develop was 2 weeks from the completion of antibiotics. Most of the repeat peritonitis cases (18 episodes, 78%) developed within 12 months from the completion of antibiotics.

Thirteen out of 140 episodes (9%) required Tenckhoff catheter removal due to refractory peritonitis (Table 5). The average time for Tenckhoff catheter removal was 9 days after the diagnosis of peritonitis. Tenckhoff catheter removal was most frequent in those who had relapsed peritonitis (27%) followed by repeat (10%) and first CoNS peritonitis (6%, overall chi-square test, *P*=0.04). Four patients underwent Tenckhoff catheter reinsertion, but only 3 patients were able to resume PD after 3 to 4 weeks of temporary HD. Another patient was converted to long-term HD due to PD technique failure. Nine patients were converted to long-term HD without Tenckhoff catheter reinsertion. Four patients (2.9%) died during the treatment of peritonitis, but only 1 death was directly attributed to CoNS peritonitis. Two other patients had sudden cardiac death and another patient died of upper gastrointestinal bleeding.

We analyzed the predictive factors of treatment response (Table 6). History of systemic antibiotic use within 3 months prior to the onset of CoNS peritonitis was associated with both a lower primary response (81% *vs* 96%, *P* < 0.01) and complete cure rate (67% *vs* 87%, *P* < 0.01). Patients with concomitant exit-site infection tend to have a lower primary response rate (69% *vs* 92%, *P* < 0.01), but the complete cure rate was similar (85% *vs* 78%, *P*=0.58). Patients with the history of CoNS peritonitis tend to have a lower primary response (82% *vs* 94%, *P*=0.03) and complete cure rate (59% *vs* 88%, *P* < 0.01). The rates of primary response and complete cure were similar between oxacillin-susceptible and oxacillin-resistant cases (91% *vs* 91%, *P*=0.90 and 76% *vs* 82%, *P*=0.45, respectively). Compared with beta-lactams, the use of vancomycin as the definitive therapy for CoNS peritonitis did not improve the complete cure rate (75% *vs* 84%, *P*=0.18). When subanalysis was performed separately on oxacillin-susceptible CoNS and oxacillin-resistant CoNS, vancomycin use (both empirical and definitive therapies) was not associated with higher rates of primary response or complete cure in either group.

On multivariate analysis, patients with concomitant exit-site infection (OR 0.06, 95% confidence interval [CI] 0.01 to 0.40, *P* < 0.01) and history of recent systemic antibiotic use (OR 0.04, 95% CI 0.01 to 0.82, *P*=0.04) were less likely to achieve primary response. Compared to first CoNS peritonitis, relapsed cases were less likely to achieve complete cure (OR 0.35, 95% CI 0.13 to 0.97, *P*=0.04). The *p* values from Hosmer–Lemeshow tests for the first and the second multivariate models were 0.38 and 0.89, respectively.

## 4. Discussion

This study is the first comprehensive examination of the frequency, predictive factors, treatment, and clinical outcomes of CoNS peritonitis among Malaysian PD patients. The overall peritonitis rate of 0.23 episodes per patient-year of treatment achieved in the current study was lower than the target recommended by ISPD [1] and was comparable to those achieved by Japan, a developed country, which reported an overall peritonitis rate of 0.22 episodes per patient-year of treatment [14].

The susceptibility pattern of CoNS isolates in the present study was similar but not identical to a recent study, which involved over 300 PD patients in Germany [5]. The authors observed a higher oxacillin resistance rate (62%) and a lower gentamicin resistance rate (30%) in the CoNS isolates compared to this present study. Antibiotic exposure promotes the emergence of antibiotic-resistant strains, whereas hospitals serve as focal points for the transmission of antibiotic-resistant bacteria [15]. Szeto and colleagues found that patients who had a history of recent hospitalization or recent systemic antibiotics had a significantly higher risk for isolation of oxacillin-resistant strains [9]. However, in our study, only patients with a history of recent hospitalization were found to have a significantly higher risk of infection with oxacillin-resistant strains. Patients may acquire oxacillin-resistant strains during the previous hospitalization through contact with healthcare workers, other patients, or contaminated objects [15].

Despite using 3-week dual antibiotic therapy, the overall primary response rate and complete cure rate in the current study were not superior compared to previous reports on CoNS peritonitis [6, 7, 9]. Gentamicin is bactericidal for CoNS species and it is particularly useful as an additional agent for oxacillin and vancomycin in prosthetic device infection [16]. Based on this rationale, gentamicin has been used routinely with beta-lactams or vancomycin in treating CoNS peritonitis in our center. However, almost half of the CoNS isolates in this study were found to be resistant to gentamicin. This could explain the nonsuperiority of the dual antibiotic therapy practiced in our center, compared to the monotherapy practiced in other centers. Perhaps, amikacin, an aminoglycoside, which tends to have a lower resistance rate among CoNS isolates, could be considered as part of the dual antibiotic therapy [17].

Nessim and colleagues also reported that having a first CoNS peritonitis episode was an independent predictive factor for an increased risk of subsequent CoNS peritonitis, with 70% of the repeat cases occurring within 1 year from the previous episode [8]. There are several possible explanations for the frequent recurrence of CoNS peritonitis. Multiple CoNS peritonitis episodes may indicate poor or nonsterile technique during PD exchange [8]. CoNS peritonitis is typically associated with the intraluminal introduction of pathogens into the peritoneal cavity. Thus, subsequent peritonitis with CoNS could be the result of biofilm formation in the peritoneal catheter. Camargo and colleagues observed that the presence of the *ica*AD gene, the gene that mediates the formation of biofilm, was associated with relapse and repeat peritonitis [7].

Choice of antimicrobials is often guided by the results obtained from antimicrobial susceptibility testing of planktonic cultured bacteria. However, this may not necessarily reflect similar susceptibilities of the same bacteria embedded in biofilms [18]. In a study comparing the antimicrobial resistance profile of planktonic CoNS and biofilm embedded CoNS, the MICs were found to be many folds higher for biofilm embedded CoNS, particularly vancomycin, where the MICs were found to be 8-fold higher. The minimal bactericidal concentrations for biofilm embedded CoNS were >256 *μ*g/mL for oxacillin, ≥128 *μ*g/mL for vancomycin, and ≥256 *μ*g/mL for gentamicin in the study [19]. These concentrations are much higher compared to the concentrations of IP antibiotics used to treat CoNS peritonitis [1]. Hence, the lower complete cure rate observed among relapsed and repeat peritonitis could partly be due to the lack of efficacy of the antibiotic therapy in eradicating CoNS embedded in the biofilm.

Similar to the findings reported by Szeto et al., vancomycin (as initial or definitive therapy) was not superior to beta-lactams in the treatment of CoNS peritonitis in our center [9]. However, Camargo et al. reported that vancomycin use as the first treatment was the independent predictive factor of resolution [7]. This could be explained by the much higher oxacillin resistance rate (70%) in their study population. The use of vancomycin as the first treatment in centers with high oxacillin resistance avoids the delay in treating oxacillin-resistant cases with an effective antibiotic, thus increasing the resolution rate. However, the use of vancomycin as definitive therapy in oxacillin-susceptible cases should not be considered as it was not superior to beta-lactam antibiotics in achieving complete cure.

In this study, history of recent systemic antibiotic use and concomitant exit-site infection were the 2 independent predictive factors identified for failure to achieve a primary response. Contrary to the findings by Szeto et al., recent hospitalization was not a predictive factor for a lower primary response rate [9]. Some studies reported that oxacillin susceptibility was an independent predictive factor of resolution for CoNS and *Staphylococcus aureus* peritonitis [7, 20], but this was not observed in this study.

Compared to monotherapy recommended by ISPD, dual antibiotic therapy using beta-lactam/gentamicin or vancomycin/gentamicin combination did not significantly improve the outcome of CoNS peritonitis. Hence, there is a need to explore other antibiotic combinations as the outcome of CoNS peritonitis remains suboptimal. *In vitro* study has demonstrated that rifampicin was effective in eradicating CoNS embedded in biofilms [21]. The use of rifampicin as an adjunct to standard antibiotic therapy in CoNS peritonitis has also been reported [22]. Hence, a randomized control trial, which compares monotherapy recommended by ISPD against dual antibiotic therapy with beta-lactam/vancomycin plus rifampicin, should be considered especially in the treatment of relapsed and repeat CoNS peritonitis.

The current study has several limitations. Firstly, species identification of CoNS was not available in the microbiology laboratory in our center. Hence, the current data do not allow the comparison of outcomes of peritonitis caused by different CoNS species. Secondly, due to the retrospective nature of the current study, the data on serum vancomycin levels were incomplete. Thus, the correlation between serum vancomycin levels and outcomes of CoNS peritonitis could not be studied. Besides, there was no documentation on the duration between patient's first presentation to the emergency department and the commencement of IP antibiotics. Thus, the effect of timely antibiotic initiation on the resolution rate of CoNS peritonitis could not be determined. Other limitations are the small number of cases and its single-center characteristic, which limit the wide extrapolation of its results.

## 5. Conclusion

Concomitant exit-site infection and history of recent systemic antibiotic use were associated with a lower primary response in CoNS peritonitis. Relapsed and repeat CoNS peritonitis were common and relapsed peritonitis was an independent predictive factor for nonresolution of CoNS peritonitis. Oxacillin resistance was common, but the treatment outcome remained favourable despite using a cefazolin-or oxacillin-based regimen as empirical therapy. The use of vancomycin in oxacillin-susceptible CoNS peritonitis should be avoided as it has not been found to improve the complete cure rate.

## Figures and Tables

**Table 1:** Patients' demographics and clinical characteristics (*N* = 95).

Characteristics	Values
Age (years, mean ± SD)	61.5 ± 10.9
Gender (male:female)	53:42
Race, *n* (%)	
Malay	67 (71%)
Chinese	23 (24%)
Indian	3 (3%)
Others	2 (2%)
Dialysis vintage at presentation, months (median (IQR))^¥^	19 (25)
PD modality, *n* (%)	
CAPD	79 (83%)
APD	15 (16%)
CCPD	1 (1%)
Diabetes mellitus, *n* (%)	77 (81%)
Recent hospitalization, *n* (%)^*∗*^	44 (46%)
Recent systemic antibiotics, *n* (%)^#^	27 (28%)
Time between symptoms onset and presentation to the hospital, days (median (IQR))^£^	2 (2)
Serum albumin at presentation, g/L (median (IQR))^§^	28 (6)
Concomitant exit-site infection, *n* (%)	9 (10%)
History of PD-related peritonitis, *n* (%)	35 (37%)

APD, automated peritoneal dialysis; CAPD, continuous ambulatory peritoneal dialysis; CCPD, continuous cycling peritoneal dialysis; IQR, interquartile range; SD, standard deviation. ^¥^The duration of time between the first day of PD commencement and the day of patients presented with signs and symptoms of PD-related peritonitis. ^*∗*^Hospitalization within 3 months before the onset of CoNS peritonitis. ^#^History of systemic antibiotic use within 3 months before the onset of CoNS peritonitis. ^£^The duration of time between the day of patients first developed symptoms of PD-related peritonitis and the day they presented to the hospital. ^§^Serum albumin level taken on the day patients first presented to the hospital with symptoms of PD-related peritonitis.

**Table 2:** Susceptibility pattern of CoNS isolates (*N* = 127).

Antibiotics	Number of resistant isolates		*P* values
	Oxacillin-susceptible (*n* (%)) *N* = 60	Oxacillin-resistant (*n* (%)) *N* = 67	
Clindamycin	5 (8%)	10 (17%)	0.11
Erythromycin	17 (25%)	43 (72%)	<0.01
Gentamicin	16 (24%)	42 (70%)	<0.01
Linezolid	0	5 (8%)	0.02
Penicillin	42 (63%)	58 (97%)	<0.01
Rifampicin	2 (3%)	9 (15%)	0.02
TMP/SMX	6 (9%)	13 (22%)	0.05

TMP/SMX, trimethoprim/sulfamethoxazole.

**Table 3:** Comparison of oxacillin and gentamicin resistance in the presence and absence of possible resistance risk factors (*N* = 127).

Possible risk factors	Resistance to oxacillin (%)		*P* values	Resistance to gentamicin (%)		*P* values
	Factor present (%)	Factor absent (%)		Factor present (%)	Factor absent (%)
Diabetes mellitus	49	42	0.57	47	42	0.70
First CoNS peritonitis^§^	43	61	0.07	41	61	0.05
Recent hospitalization^*∗*^	56	38	0.04	55	36	0.04
Recent systemic antibiotics^#^	55	43	0.16	57	39	0.04
Serum albumin ≤30 g/L^£^	50	39	0.29	48	39	0.40

^§^Patients had no history of CoNS peritonitis before the current episode of peritonitis. ^*∗*^Hospitalization within 3 months before the onset of CoNS peritonitis. ^#^History of systemic antibiotic use within 3 months before the onset of CoNS peritonitis. ^£^Serum albumin level taken on the day patients first presented to the hospital with symptoms of PD-related peritonitis.

**Table 4:** Antimicrobial agents prescribed for CoNS peritonitis (*N* = 140).

Antibiotics	First peritonitis (*N* = 96)		Relapsed peritonitis (*N* = 15)		Repeat peritonitis (*N* = 29)		Overall (*N* = 140)	
	Empirical therapy	Definitive therapy	Empirical therapy	Definitive therapy	Empirical therapy	Definitive therapy	Empirical therapy	Definitive therapy
Gram-positive coverage								
Cefazolin	65 (68%)	18 (19%)	3 (20%)	0	20 (69%)	7 (24%)	88 (63%)	25 (18%)
Cloxacillin	27 (28%)	24 (25%)	3 (20%)	2 (13%)	5 (17%)	3 (10%)	35 (25%)	29 (21%)
Vancomycin	2 (2%)	53 (55%)	8 (53%)	11 (74%)	4 (14%)	19 (66%)	14 (10%)	83 (59%)
Others^*∗*^	2 (2%)	1 (1%)	1 (7%)	2 (13%)	0	0	3 (2%)	3 (2%)
Gram-negative coverage								
Ceftazidime	94 (98%)	NA	11 (73%)	NA	28 (97%)	NA	133 (95%)	NA
Aminoglycosides								
Gentamicin	92 (96%)	81 (84%)	14 (93%)	14 (93%)	27 (93%)	25 (86%)	133 (95%)	120 (86%)
Amikacin	3 (3%)	12 (13%)	0	0	2 (7%)	3 (11%)	5 (4%)	15 (11%)
Not given	1 (1%)	3 (3%)	1 (7%)	1 (7%)	0	1 (3%)	2 (1%)	5 (3%)

^*∗*^Others include cefepime, linezolid, meropenem, piperacillin/tazobactam, rifampicin, and trimethoprim/sulfamethoxazole. NA, not applicable as ceftazidime was discontinued once CoNS was isolated from peritoneal dialysate samples.

**Table 5:** Outcomes of CoNS peritonitis.

Outcomes of CoNS peritonitis	Types of CoNS peritonitis (*N* = 140)			*P* values (overall chi-square test)
	First (*n* = 95)	Relapsed (*n* = 15)	Repeat (*n* = 30)	
Primary response	89 (94%)	11 (73%)	26 (87%)	0.04
Complete cure	83 (87)	7 (47)	20 (67)	<0.01
Relapsed^*∗*^	7 (8%)	4 (33%)	6 (25%)	0.01
Repeat^*∗*^	18 (20%)	1 (8%)	4 (17%)	0.60
Catheter removal	6 (6%)	4 (27%)	3 (10%)	0.04
Conversion to long-term HD	5 (5%)	3 (20%)	2 (7%)	0.13
Death	1 (1%)	0	3 (10%)	0.03

CoNS, coagulase-negative *Staphylococci*; HD, haemodialysis. ^*∗*^Excluded cases who passed away or were converted to HD permanently due to refractory peritonitis (126 cases were included for analysis).

**Table 6:** Predictive factors for primary response and complete cure (*N* = 140).

Predictive factors	Achieved primary response (%)		*P* values	Achieved complete cure (%)		*P* values
	Factor present	Factor absent		Factor present	Factor absent
Beta-lactam antibiotics as empirical therapy	91%	79%	0.13	83%	43%	<0.01
Beta-lactam antibiotics as definitive therapy	NA	NA	NA	84%	75%	0.18
Concomitant exit-site infection	69%	92%	<0.01	85%	78%	0.58
Diabetes mellitus	91%	87%	0.49	78%	80%	0.83
First CoNS peritonitis	94%	82%	0.03	88%	59%	<0.01
Recent hospitalization	86%	95%	0.06	74%	84%	0.13
Recent systemic antibiotics	81%	96%	<0.01	67%	87%	<0.01
Serum albumin ≤30 g/L	88%	97%	0.09	76%	86%	0.20
Susceptible to oxacillin^*∗*^	91%	92%	0.90	76%	82%	0.45

^*∗*^Only 127 cases had antibiotics susceptibility data reported. NA: not applicable.

## Data Availability

The data used to support the findings of this study are available from the corresponding author upon request.
